# Porcine low-density lipoprotein receptor plays an important role in classical swine fever virus infection

**DOI:** 10.1080/22221751.2024.2327385

**Published:** 2024-03-21

**Authors:** Elena Leveringhaus, Robin Poljakovic, Gina Herrmann, Gleyder Roman-Sosa, Paul Becher, Alexander Postel

**Affiliations:** Institute of Virology, University of Veterinary Medicine Hannover, Hannover, Germany

**Keywords:** Classical swine fever virus, CSFV, Bungowannah pestivirus, low-density lipoprotein receptor, LDLR, *Flaviviridae*

## Abstract

Several cellular factors have been reported to be required for replication of classical swine fever virus (CSFV), a member of the genus *Pestivirus* within the family *Flaviviridae*. However, many steps of its replication cycle are still poorly understood. The low-density lipoprotein receptor (LDLR) is involved in cell entry and post-entry processes of different viruses including other members of the *Flaviviridae*. In this study, the relevance of LDLR in replication of CSFV and another porcine pestivirus, Bungowannah pestivirus (BuPV), was investigated by antibody-mediated blocking of LDLR and genetically engineered porcine cell lines providing altered LDLR expression levels. An LDLR-specific antibody largely blocked infection with CSFV, but had only a minor impact on BuPV. Infections of the genetically modified cells confirmed an LDLR-dependent replication of CSFV. Compared to wild type cells, lower and higher expression of LDLR resulted in a 3.5-fold decrease or increase in viral titers already 20 h post infection. Viral titers were 25-fold increased in LDLR-overexpressing cells compared to cells with reduced LDLR expression at 72 h post infection. The varying LDLR expression levels had no clear effect on permissivity to BuPV. A decoy receptor assay using recombinant soluble LDLR provided no evidence that LDLR may function as a receptor for CSFV or BuPV. Differences in their dependency on LDLR suggest that CSFV and BuPV likely use different mechanisms to interact with their host cells. Moreover, this study reveals similarities in the replication cycles of CSFV and other members of the family *Flaviviridae* that are dependent on LDLR.

## Introduction

The steadily growing genus *Pestivirus* belonging to the family *Flaviviridae* currently comprises 19 approved species [1-3]. Pestiviruses have a positive-sense single-stranded RNA genome that encodes the viral capsid protein, three envelope glycoproteins (E^rns^, E1 and E2) and eight non-structural proteins [4].

The majority of the pestiviruses known to date infect cloven-hoofed animals, and these infections can have severe consequences for the health status of the host, e.g. immunosuppression, reproductive disorders or haemorrhagic fever. Classical swine fever virus (CSFV, species *Pestivirus suis*) is one of the most important pathogens of pigs [5]. There are three major genotypes with numerous sub-genotypes comprising CSFV strains of varying virulence. Infections with highly virulent strains can lead to high mortality rates, especially in young pigs. Due to its enormous socio-economic consequences, classical swine fever (CSF) is a notifiable animal disease [6].

Associated with an outbreak of sudden deaths in young piglets and an increase in stillbirths and mummified fetuses on two farms in Australia, a genetically distinct pestivirus was described in 2007 and named Bungowannah pestivirus (BuPV, species *Pestivirus australiaense*) [7]. The clinical presentation was referred to as the porcine myocarditis (PMC) syndrome. At times, piglet losses exceeded 50% [8]. Since its discovery, other pestiviruses genetically related to BuPV have been identified, namely, the LINDA virus (LindaV) isolated from diseased piglets showing congenital tremor and the phocoena pestivirus (PhoPeV) isolated from harbour porpoises [9,10].

Pestiviruses enter their host cells via receptor-mediated endocytosis [11,12]. Bovine complement regulatory protein 46 (CD46) has been shown to be an important cellular receptor for the bovine pestiviruses bovine viral diarrhoea virus types 1 and 2 (BVDV-1 and -2, species *Pestivirus bovis* and *Pestivirus tauri*) and HoBi-like pestivirus (HoBiPeV, species *Pestivirus brazilense*), but not for giraffe pestivirus (GPeV, species *Pestivirus giraffae*) which also efficiently replicates in bovine cells [13-15]. Porcine CD46 has been suggested to be an entry factor for CSFV, but a recent study revealed that CSFV as well as BuPV are independent of CD46, while it is on the other hand an important receptor for the highly distinct atypical porcine pestivirus (APPV, species *Pestivirus scrofae*) [16,17].

Small interfering RNA (siRNA)-mediated knockdown of the low-density lipoprotein receptor (LDLR) suggested that this membrane protein might play a role in CSFV infection [18]. LDLR belongs to the LDLR family responsible for cholesterol homeostasis [19]. It regulates the plasma cholesterol levels by endocytosis of cholesterol-rich low-density lipoproteins (LDL) which occurs mainly in the liver. Still, it is widely expressed in almost all tissues and organs in humans. The ectodomain of LDLR includes the ligand-binding domain (LBD) which consists of seven repeats (L1-L7) and the epidermal growth factor precursor homology domain (EGFPH) [20]. LDLR not only plays an important role in lipid metabolism and thus in various metabolic disorders, but it is also exploited by several viruses in different stages of their life cycle [21-23].

Within the family *Flaviviridae*, the hepacivirus hepatitis C virus (HCV), which is of outstanding importance in human medicine, uses LDLR as an entry factor [24,25]. As HCV replication, assembly and egress are intertwined with the cellular lipid metabolism, LDLR is also involved in later stages of the HCV life cycle [26]. Moreover, highly relevant (re-)emerging orthoflaviviruses such as dengue virus (DENV) and Zika virus (ZIKV) strongly depend on cholesterol whose cellular uptake is to a large extent regulated via LDLR [27]. The role of LDLR in entry of BVDV has been discussed contradictorily. Using the same cell line resistant to BVDV infection and the same anti-LDLR antibodies, one study concluded that BVDV uses LDLR as a receptor, while another study reported opposite results [25,28]. Very recently, it was discovered that the non-permissive phenotype of this cell line is due to the lack of ADAM17 [29].

In this study, we investigated the relevance of LDLR in infection with the porcine pestiviruses CSFV and BuPV. For this purpose, antibody-mediated blocking of LDLR as well as *in vitro* infection experiments with genetically modified porcine cells providing reduced and enhanced levels of LDLR were performed. The results show that availability of LDLR plays an important role in CSFV infection, while it has a less clear impact on viral replication of BuPV. A decoy receptor assay provided no evidence that soluble LDLR is able to neutralize CSFV or BuPV infection. Nevertheless, parallels to related viruses within the family *Flaviviridae* may open new avenues towards a better understanding of the virus-host interactome in entry and post-entry processes of LDLR-dependent viruses.

## Material and methods

### Cells

The porcine kidney cell line PK15 (cell line 5-1) and porcine embryonic kidney cell line SPEV (cell line 0008) originated from the Friedrich Loeffler Institute (FLI, Greifswald – Insel Riems, Germany). PK15 and SPEV cells were cultured in Eagle’s Minimum Essential Medium (EMEM) supplemented with 1000 U/ml penicillin, 50 µg/ml streptomycin and either 10% (PK15) or 5% (SPEV) fetal bovine serum (FBS) free of pestivirus genomes and pestivirus-specific antibodies. The human embryonic kidney (HEK) cell line 293 T (ACC 635) originating from the Leibniz Institute (DSMZ – German Collection of Microorganisms and Cell Cultures, Braunschweig, Germany) was maintained in Dulbecco’s Modified Eagle’s Medium (DMEM) including antibiotics and 10% FBS. All cell cultures were incubated at 37°C in a humidified atmosphere containing 5% CO2.

### Viruses

The classical swine fever virus (CSFV) isolates Alfort-Tübingen (AlfT, CSF0904, genotype 2.3), Riems (CSF0913, genotype 1.1) and Kozlov (CSF0382, genotype 1.1) came from the virus collection of the European Union (EU) and World Organisation for Animal Health (WOAH) Reference Laboratory for Classical Swine Fever, Institute of Virology, University of Veterinary Medicine Hannover, Hannover, Germany; CSF catalogue numbers in parentheses [30].

Infectious CSFV AlfT was recovered from PK15 cells transfected with *in vitro*-transcribed RNA derived from the full-length cDNA clone pAlfort-p2447 of CSFV strain Alfort-p447 [32,33]. While CSFV strain AlfT shows a moderate virulence, CSFV strain Riems, a variant of the lapinized C-strain, is an attenuated vaccine strain. In contrast, CSFV strain Kozlov is characterized by an extraordinarily high virulence and is often used in vaccine challenge studies.

Bungowannah pestivirus (BuPV) was kindly provided by Peter Kirkland (Elizabeth Macarthur Agricultural Institute, Menangle, Australia) [7].

Recombinant replication-deficient vesicular stomatitis virus (VSV) harbouring the sequence of enhanced Green Fluorescent Protein (eGFP) instead of the sequence encoding for the G protein (VSVΔG-G(eGFP/fLuc), here referred to as rVSV) was generated as described before using the plasmid system and packaging cell line kindly provided by Gert Zimmer (Institute of Virology and Immunology, Mittelhäusern, Switzerland) [34].

### Detection of porcine LDLR by Western Blot

Cells were seeded in 6-well-plates and 10 cm dishes and grown to confluence. Before harvesting, the cell count was determined. Cells in 6-well-plates were washed three times with cold phosphate-buffered saline (PBS), lysed by NP40 lysis buffer and harvested using a cell scraper. Cells in 10 cm dishes were submitted to membrane protein extraction using the Mem-PER^TM^ Plus Membrane Protein Extraction Kit (89842, Thermo Scientific) according to the manufacturer’s instructions. Lysed cells and solubilized membrane proteins were stored at −80°C.

Based on the cell count, adjusted sample amounts were subjected to SDS-PAGE under reducing conditions and subsequent Western Blotting. After blocking with Tris-buffered saline (TBS) + 0.02% Tween + 5% milk powder, LDLR was detected using either a monoclonal LDLR antibody (ab52818, Abcam; diluted 1:1,000 in blocking buffer) or a polyclonal LDLR antibody (10785-1-AP, Proteintech; diluted 1:2,000), followed by StarBright™ Blue 520 Goat Anti-Rabbit IgG secondary antibody (12005870, Bio-Rad; diluted 1:2,500). The housekeeping protein beta-Actin was stained by a monoclonal beta-Actin antibody (AM4302, Invitrogen; diluted 1:1,000) in combination with StarBright Blue 700 Goat Anti-Mouse IgG secondary antibody (12004159, Bio-Rad; diluted 1:2,500). Membrane protein preparations were additionally stained for the plasma membrane marker protein Ezrin using a monoclonal Ezrin antibody (SAB4200806, Sigma-Aldrich; diluted 1:500) in combination with the same secondary antibody. Blots were analyzed using the Bio-Rad ChemiDoc MP Imaging System and Image Lab software (version 6.1.0). For quantitative comparison of the LDLR expression of different cell lines, beta-Actin or Ezrin were used for normalization.

### Detection of porcine LDLR by immunofluorescence microscopy

Cells were grown in 24-well-plates to reach confluence after 48 h. Following a washing step with PBS, cells were heat-fixed at 80°C for 4 h. For staining of LDLR, cells were incubated with either the monoclonal LDLR antibody (ab52818, Abcam; diluted 1:500 in PBS + 0.05% Tween) or the polyclonal LDLR antibody (10785-1-AP, Proteintech; diluted 1:500) at 37°C for 1 h, followed by three washing steps and incubation with secondary antibody goat anti-rabbit IgG-Cy3 (111-165-003, dianova; diluted 1:1,000) at 37°C for 1 h. After another three washes, cell nuclei were stained with 4’,6-diamidino-2-phenylindole (DAPI; D3571, Thermo Fisher Scientific; diluted 1:500).

For immunofluorescence (IF) analysis, a Leica DMI3000 B microscope with a Leica DFC3000 G camera (Leica Microsystems) and the Leica Application Suite software (version 4.13.0) were employed. For quantitative comparison of the LDLR expression of different cell lines, the experiment was repeated three times, and each time five pictures per well were taken. Using the ImageJ software (version 1.51q), images were converted to HSB stacks and the total brightness was measured. The average brightness per cell was calculated using the DAPI staining as reference.

### Antibody-mediated blocking of LDLR

In order to achieve LDLR-deficiency, cellular LDLR was blocked using the polyclonal LDLR antibody (10785-1-AP, Proteintech). A non-relevant antibody (anti-CD45, 60287-1-Ig, Proteintech) from the same manufacturer, dissolved in the same storage buffer, served as control.

One day prior to infection, cells were seeded in 96-well-plates to reach confluence at 20 h post infection (hpi). On the day of infection, each cell line was trypsinized and the cell count was determined. Cells were washed once with PBS and incubated with either LDLR or control antibody diluted in 50 µl cell culture media at 37°C for 1 h. Subsequently, the antibody dilutions were discarded and cells were washed twice with PBS. Virus dilutions with multiplicity of infection (MOI) = 1 for CSFV strains AlfT, Riems and Kozlov and MOI = 0.5 for the highly efficiently replicating BuPV were prepared in 50 µl cell culture media. Cells were inoculated with the virus dilutions and incubated at 37°C on a rocking platform for 2 h. Subsequently, virus inocula were discarded and the cells were washed twice with PBS. Finally, 100 µl fresh media containing the same amount of antibody as used beforehand was added followed by further incubation. At 20 hpi, supernatants were harvested and stored at –80°C for later virus titration. Cells were washed once with PBS, dried and heat-fixed at 80°C for 4 h.

In order to examine whether the LDLR antibody affected virus particle activity, virus inocula were pre-incubated with either LDLR or control antibody at 37°C for 1 h, or virus inocula and the respective antibody were separately added to the cells at the same time.

### Genetic engineering of porcine cells

In order to establish a stable LDLR-deficiency, the generation of a porcine LDLR-knockout (KO) cell line was attempted by CRISPR/Cas9 genome editing as described in a previous study [14] with the following modifications: Two strategies with different guide RNA target sequences of 20 nucleotides (nt), respectively, one close to the 5’ end within the region encoding for the signal peptide (SP) and one at the beginning of exon 3 encoding for repeat L2 of porcine LDLR were pursued (strategy 1 shown in Figure 2). The lentiviral particles produced with the lentiviral transfer vector pLentiCRISPR-v2_LDLRpig containing the corresponding complementary oligonucleotides gLDLRpig-1_fw and gLDLRpig-1_rev or gLDLRpig-2_fw and gLDLRpig-2_rev (Table 1) were used to transduce PK15 and SPEV cells. After two rounds of biological cloning, a clonal cell line was obtained as proven by genetic characterization as described below.

**Table 1.** Oligonucleotides / primers used in this study.

Name	Sequence (5’ → 3’)	Target	Application
gLDLRpig-1_fw	CACCGGCAAGGCGACAGCCCATCGC	SP	Generation of LDLR-knockout cell line
gLDLRpig-1_rev	AAACGCGATGGGCTGTCGCCTTGCC		
gLDLRpig-2_fw	CACCGGTCTGTCACCTGCAAGATAG	LBD-L2	Generation of LDLR-knockout cell line
gLDLRpig-2_rev	AAACCTATCTTGCAGGTGACAGACC		
LDLRpig_93-fw	GCACACAGTGAACAGGAGCC	exon 1	Genetic characterization of LDLR-knockout cell line
LDLRpig_333-rev	CACGTCTCCAGGGACTCATC	LBD-L1	

Notes: underlined: CRISPR/Cas9 guide RNA sequence; exon 1: exon 1 of the LDLR gene; SP: sequence encoding for the signal peptide of LDLR; LBD-L1 / L2: sequence encoding for the first / second repeat of the ligand binding domain of LDLR.

Furthermore, this cell line was trans-complemented with porcine LDLR using recombinant lentiviruses, following the same principle as described previously [14]. Briefly, RNA was isolated from SPEV WT cells, transcribed into cDNA and the LDLR encoding sequence was amplified by proofreading polymerases to obtain a DNA insert for cloning. The CRISPR/Cas9 target sequence used to generate the LDLR-KO cell line was modified by introduction of four silent mutations (Figure 2). The complete LDLR encoding sequence was cloned into the lentiviral plasmid vector pWPI-msc-GUN [35] in several steps. Details of the cloning strategy and primers used are available upon request. Production of lentiviral particles, lentiviral transduction and selection of transduced cells were performed as described before [14].

### Genetic characterization of engineered porcine cells

CRISPR/Cas9 edited cell lines were genetically characterized by PCR using primer pairs flanking the respective guide RNA target sequence and Sanger sequencing of PCR amplicons. The genome alterations of one cell line were characterized in detail by Sanger sequencing of cloned cDNA fragments derived from mRNA isolated from the cells as described previously [14]. As templates for sequencing, 241 bp amplicons were generated using primers LDLRpig_93-fw and LDLRpig_333-rev (Table 1). Sequences from 20 plasmids were analyzed and genetic alterations were identified by comparison with the LDLR consensus sequence obtained from the parental cell line.

Sequence alterations were additionally analyzed using the online prediction tools SignalP – 6.0 [36,37] and PrediSi [38,39].

### Low-density lipoprotein uptake assay

Wild type (WT) and genetically modified cell lines were seeded in 24-well-plates one day prior to the experiment, conducted as described previously [28]. Cells were deprived of FBS by removing the cell culture media and adding EMEM without FBS for 4 h, before adding 10 µg/ml fluorescent-labelled DiI-LDL (L3482, Invitrogen). After incubation at 37°C for 1 h, cells were washed three times with PBS and DiI-LDL uptake was immediately evaluated by IF analysis.

Again, five pictures per well were taken and the brightness was measured. For calculation of the average brightness per cell, the respective cell number was determined by counting of trypsinized cells directly prior to addition of DiI-LDL.

### Infections of genetically engineered porcine cell lines

One day prior to infection, cells were seeded in 24-well-plates to reach confluence at either 20 or 72 hpi. After determination of the cell numbers and disposal of the cell culture media, the cells were infected as described above with virus dilutions prepared in 500 µl media and incubated at 37°C on a rocking platform. After 2 h, virus inocula were discarded and cells were washed three times with PBS. After addition of 1 ml fresh cell culture media, cells were incubated for additional 20 or 72 h. At 20 or 72 hpi, supernatants were harvested and stored at −80°C and cells were treated and heat-fixed as described above. All infection experiments were repeated three times.

### Expression and purification of soluble porcine LDLR

The sequence encoding for the porcine LDLR ectodomain comprising amino acids (aa) E57 to S804 (Accession no. XP_020936108) was cloned into a pCAGGS expression plasmid. In the resulting recombinant fusion protein, the LDLR ectodomain was N-terminally fused with the aa sequence MGILPSPGMPALLSLVSLLSVLLMGCVAETG representing the signal peptide of the spike glycoprotein of middle east respiratory syndrome-related coronavirus (MERS-CoV) for secretion and C–terminally fused with the aa sequence SGLNDIFEAQKIEWHESGGGSLVPRGS**WSHPQFEK**GGGSGGGSGGGS**WSHPQFEK** containing a Twin Strep peptide tag (bold) used for purification [40]. This soluble variant of porcine LDLR (sLDLR) was produced following a method optimized for recombinant protein expression in transiently transfected HEK 293 T cells [41]. Supernatants were harvested 72 h after transfection and sLDLR was affinity-purified essentially as described previously [40] using a Strep-Tactin XT Sepharose matrix (29401317, cytiva) according to the manufacturer's instructions on an ÄKTA^TM^ pure – protein purification system (GE Life Sciences). Protein fractions containing recombinant sLDLR were quantified spectro-photometrically and stored at -80°C. Before use, the fractions were pooled and subjected to Western Blot analysis. Recombinant sLDLR was detected either directly using the aforementioned polyclonal LDLR antibody or indirectly using a Strep-Tactin HRP conjugate (2-1502-001, iba) as instructed by the manufacturer.

### sLDLR decoy receptor assay

One day prior to infection, PK15 cells were seeded in 96-well-plates to reach confluence at the time point of IF analysis. After cell counting, virus dilutions with MOI = 1 for CSFV strain Kozlov and MOI = 0.5 for BuPV were prepared in 30 µl media. Virus dilutions were pre-incubated with a pool of purified sLDLR fractions containing approx. 16.6 µg total protein diluted in serum free media to a volume of 150 µl at 37°C for 30 min before inoculation of the cells. After 2 h, infected cells were washed three times with PBS and 100 µl fresh media containing 7.8 µg of the purified sLDLR was added. Cells were heat-fixed at 20 hpi as described before.

The decoy receptor assay was established with VSV, which is known to use LDLR as receptor. For this purpose, rVSV expressing eGFP was used. To monitor cell entry after rVSV inoculation (MOI = 1), infected PK15 cells were detected by their green fluorescence performing live cell imaging at 5 hpi. To exclude that the C-terminally located Twin Strep tag was capable to neutralize viral activity, a control peptide (Strep-tag II; 2-1018-002, iba) was added separately according to the same procedure.

### Immunofluorescence analysis

Pestivirus infections were visualized by indirect IF staining. CSFV was stained using monoclonal antibody C16, which was raised against BVDV non-structural protein NS3 and is commonly used for detection of different pestiviruses including CSFV (diluted 1:50), in combination with secondary antibody goat anti-mouse IgG-Cy3 (115-165-146, dianova; diluted 1:800). After the LDLR blocking experiment, isotype-specific secondary antibody goat anti-mouse IgG1-Cy3 (115-165-205, dianova; diluted 1:800) was used. Staining of BuPV was performed by incubation with a porcine BuPV-specific antiserum (kindly provided by Peter Kirkland, Elizabeth Macarthur Agricultural Institute, Menangle, Australia; diluted 1:10,000) at 4°C overnight, followed by secondary antibody goat anti-swine IgG-Alexa Fluor 594 (114-585-003, dianova; diluted 1:1,000). Cell nuclei were stained with DAPI (D3571, Thermo Fisher Scientific; diluted 1:500).

In addition to qualitative evaluation by IF microscopy, infections were quantified using the ImageJ software. Therefore, five pictures per well were converted to binary, and positive pixels were counted. In order to ensure comparability of the different cell lines, data were normalized against the respective amount of cells using the DAPI staining.

### Flow cytometry

In addition to IF analysis, CSFV strain Kozlov infection of genetically modified cell lines was evaluated by flow cytometry (FC). For this purpose, the infection was conducted as described above with MOI = 5 for infections with 20 h incubation time. At 20 or 72 hpi, cells were washed, detached, fixed with 4% paraformaldehyde (PFA) and stained using antibody C16 (diluted 1:25 in PBS + 0.1% Triton X100) in combination with secondary antibody goat anti-mouse IgG-Alexa Fluor 647 (115-605-003, dianova; diluted 1:400) (details available upon request). Samples were analyzed on a Miltenyi MACSQuant Analyzer 10 and data were evaluated using the FlowLogic software (version 8.7).

### Virus titration

Infections were evaluated and quantified by virus titration of the supernatants harvested before fixation. Viral titers were determined by end-point dilution (with five-fold dilutions) in quadruplicates on PK15 cells. Cells were heat-fixed after 72 h and titrations were analyzed by IF staining performed as described for the infection experiments. The 50% tissue culture infective dose per ml (TCID_50_/ml) was calculated using the Spearman-Kaerber method [42,43].

### Statistical analysis

Data were prepared with Microsoft Excel and statistically analyzed using the GraphPad Prism software (version 9.0.0). For analysis of the antibody blocking experiment, *p*-values were calculated by unpaired t-tests with Welch's correction. Significance of differences in LDLR expression was calculated by Brown-Forsythe and Welch ANOVA tests with Dunnett's T3 multiple comparisons correction. For LDL uptake as well as IF analysis of infections of engineered cell lines and the sLDLR decoy receptor assay, Kruskal–Wallis test with Dunn's multiple comparisons correction was used. Significance of differences in viral titers obtained from infected engineered cell lines was calculated by ordinary one-way ANOVAs with Tukey’s multiple comparisons correction. Differences between groups were interpreted as non-significant, significant (*p < 0.05), very significant (**p < 0.01) or extremely significant (***p < 0.001).

## Results

### Detection and expression of LDLR in cell lines permissive to CSFV

According to the manufacturers, both LDLR antibodies used in this study were generated against human LDLR and predicted to cross-react with porcine LDLR. Our results confirm that both antibodies bind to porcine LDLR and are suitable for LDLR detection in both Western Blot and IF (Figure S1). However, the monoclonal antibody showed low sensitivity for the membrane protein preparations, likely due to its C-terminal epitope location (data not shown). In contrast, membrane-bound LDLR was well detectable with the polyclonal antibody whose immunogen (aa 1–350 of human LDLR) includes the complete ligand-binding domain (LBD) of LDLR.

Western Blot and IF microscopy showed that both cell lines used in this study, PK15 and SPEV, express LDLR (Figure S1). Western Blot detection of LDLR in membrane protein preparations indicated that LDLR is present on the cell surface. A band size of approx. 160 kDa was observed as also described by other authors [44,45]. In addition to the mature form, a second band with a size of approx. 120 kDa likely representing a precursor of LDLR was detected (Figure S1, panel A) [44,45]. In IF microscopy, both PK15 and SPEV cells and both antibodies showed a similar specific staining (Figure S1, panel B).

### Impact of LDLR-deficiency by antibody-mediated blocking on infection with porcine pestiviruses

In order to investigate the relevance of LDLR in infection with different porcine pestiviruses, antibody-mediated blocking of LDLR on the surface of PK15 and SPEV cells was performed.

For CSFV, blocking of LDLR on PK15 as well as SPEV cells resulted in a clear, dose-dependent reduction in the number of infected cells. The effect was more pronounced on PK15 cells, likely due to their higher permissivity to CSFV. Due to its high replication rate, the number of infected cells at 20 hpi was highest for CSFV strain Kozlov. Thus, infections of PK15 cells with CSFV strain Kozlov are shown (Figure S2, panel A). For BuPV, 10 µg of the anti-LDLR antibody also resulted in a strongly reduced infection whereas 5 µg had hardly any effect (Figure S2, panel A). Since the cells treated with 10 µg anti-LDLR antibody showed reduced growth and abnormal morphology, further blocking experiments were carried out using 5 µg of the anti-LDLR antibody. Pre-incubation of virus inocula with 5 µg anti-LDLR antibody as well as separate, simultaneous addition of virus inocula and antibody to the cells resulted in a slight reduction of CSFV, but not BuPV infection in comparison to the control antibody (Figure S2, panel B). Thus, there is no indication of an effect of the anti-LDLR antibody on virus particle activity, but of a rapid reaction of unbound antibody with cellular LDLR.

**Figure 1.**
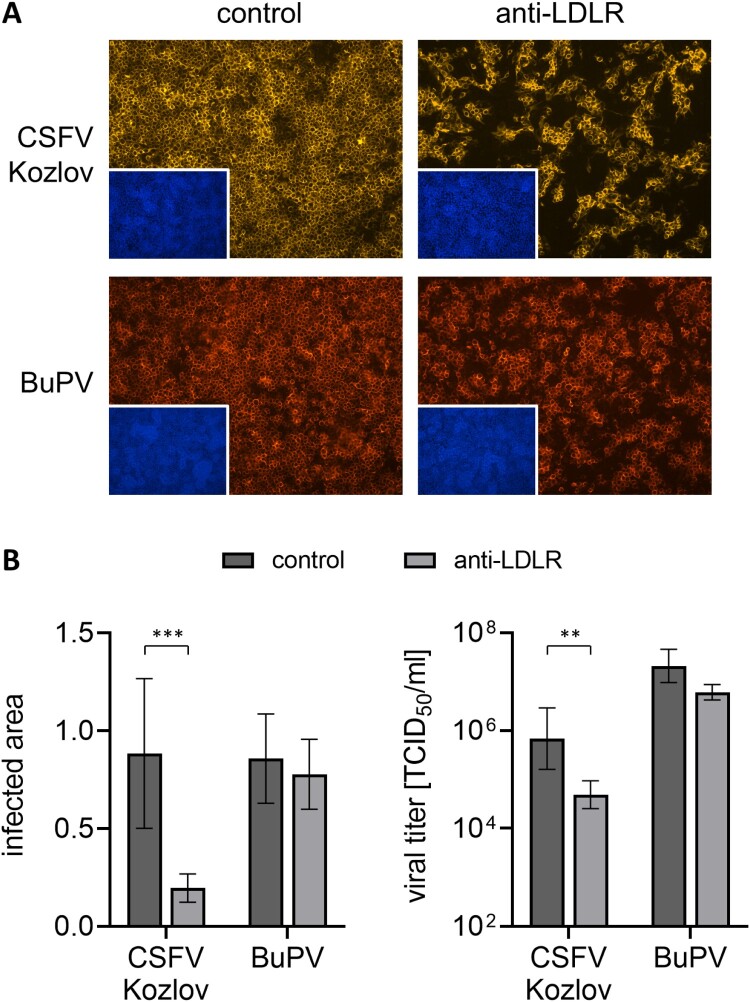
Impact of LDLR-blocking on infection with porcine pestiviruses. (A) PK15 cells were pre-incubated with either anti-LDLR polyclonal antibody (anti-LDLR) or non-relevant anti-CD45 antibody (control) and infected with CSFV strain Kozlov or BuPV. After infection, the respective antibody was added again for the incubation period of 20 h. Infections were evaluated by immunofluorescence (IF) staining of pestiviruses using mab C16 for CSFV or porcine BuPV-specific antiserum, respectively, in combination with either isotype-specific anti-mouse IgG1 (orange) or anti-swine (red) secondary mab. Nuclei were stained with DAPI (blue) to confirm the presence of confluent monolayers (small pictures in lower left corners). Representative pictures from three independent experiments are shown. (B, left) Infections were quantified as infected areas in IF by pixel counting using ImageJ. Orange/red pixels (detected in virus-positive cells) were normalized against blue pixels (detected in cell nuclei). Bars represent mean values from 15 pictures comprising five pictures from each of the three independent experiments, respectively. Standard deviations are indicated. (B, right) Infectious viral titers in the supernatants from infected cells were determined by endpoint dilution assay in quadruplicates. Bars represent mean 50% tissue culture infectious doses per ml (TCID_50_/ml) from three independent experiments. Standard deviations are indicated. (B) Significance of differences was calculated by unpaired t-tests with Welch's correction (*p < 0.05, **p < 0.01, ***p < 0.001).

IF analysis of blocking experiments showed that the number of PK15 cells infected with CSFV strain Kozlov was reduced by 78% after blocking of LDLR with 5 µg anti-LDLR antibody (***p < 0.001). Accordingly, viral titers obtained from these cells were strongly reduced (approx. 21-fold; **p < 0.01) after blocking of LDLR (Figure 1). Although replication of CSFV strains AlfT and Riems was less efficient, comparable effects were also observed for these two distinct strains. At 20 hpi, infections of LDLR-blocked PK15 cells with CSFV strains AlfT and Riems were reduced by 93% and 86% in comparison to the infections of control cells, respectively (data not shown).

For BuPV, IF analysis showed a non-significant minor reduction of 9% in infection of PK15 cells after blocking with 5 µg anti-LDLR antibody. The difference in the viral titers was also less pronounced (approx. 5-fold) compared to CSFV (Figure 1).

Albeit viral replication was overall lower on SPEV cells, similar results as obtained with PK15 cells were observed using SPEV cells for both CSFV and BuPV, respectively (Figure S2, panel C).

### Genetic characterization of engineered porcine cells

In order to further investigate the LDLR dependency of CSFV and BuPV suggested by antibody-mediated blocking, CRISPR/Cas9 genome editing was performed to generate a LDLR-KO cell line. The genomic alterations of six different engineered cell lines were examined by Sanger sequencing of PCR amplicons from the LDLR encoding region comprising the respective guide RNA target sequence. The sequences revealed that none of the PK15 cell lines showed alterations in the LDLR encoding sequence. The SPEV cell lines showed alterations, but none of them contained a functional LDLR knockout. Based on the results of this screening, the cell line containing the largest deletion in the LDLR encoding sequence was selected for biological cloning and further genetic characterization.

**Figure 2.**
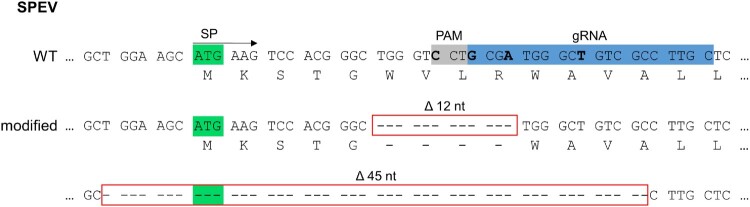
Genome editing strategy and genetic characterization of engineered embryonic porcine kidney cells (SPEV). In the top rows, the LDLR consensus nucleotide and amino acid sequences present in SPEV wild type (WT) cells are depicted. The first 45 nucleotides (nt) of the sequence encoding for the signal peptide (SP; total length: 66 nt) including the start codon (highlighted in green) as well as the position of the CRISPR/Cas9 guide RNA (gRNA, highlighted in blue) and its protospacer adjacent motif (PAM, highlighted in grey) are shown. Below, the nucleotide and deduced amino acid sequences obtained from the genetically engineered (modified) cell line are given. Deletions of 12 and 45 nt (Δ12 nt, Δ45 nt) are boxed in red. For trans-complementation of LDLR, the gRNA and PAM sequence were altered by introduction of four silent mutations (nucleotides to be mutated highlighted in bold).

Genetic characterization was performed by analysis of individual recombinant plasmids containing PCR amplicons obtained from mRNA transcripts of the LDLR encoding region targeted by genome editing. Sequence analysis of 20 plasmids confirmed the presence of two different mRNA variants transcribed from the diploid LDLR encoding gene locus. One allele exhibited a deletion of 45 nucleotides including the start codon of the SP, leading to no translation of the LDLR encoding sequence from this allele. The other allele held an in-frame deletion of 12 nucleotides, resulting in the absence of the sixth to ninth aa residues of the SP. The shortened SP was predicted to be cleaved from the following LDLR protein at the correct position and to still function as an SP by both prediction tools, SignalP – 6.0 and PrediSi. However, cleavage probability and score were predicted to be approx. 5% (SignalP – 6.0) and approx. 14% (PrediSi) lower in comparison to the native SP of the SPEV WT cells, respectively. The analysis of the two sequences indicated the successful generation of a heterozygous LDLR^+/-^ cell line (Figure 2).

The LDLR encoding sequence obtained from SPEV cells used for trans-complementation of the heterozygous LDLR^+/-^ cell line was analyzed to encode for a protein variant identical to a porcine LDLR aa sequence deposited in GenBank (Accession no. XP_020936108). Sequence analysis confirmed the presence of four silent mutations in the gRNA and PAM sequence that were introduced to avoid unintended cleavage by the Cas9 enzyme (Figure 2).

### Phenotypic characterization of engineered porcine cells

#### LDLR expression

**Figure 3.**
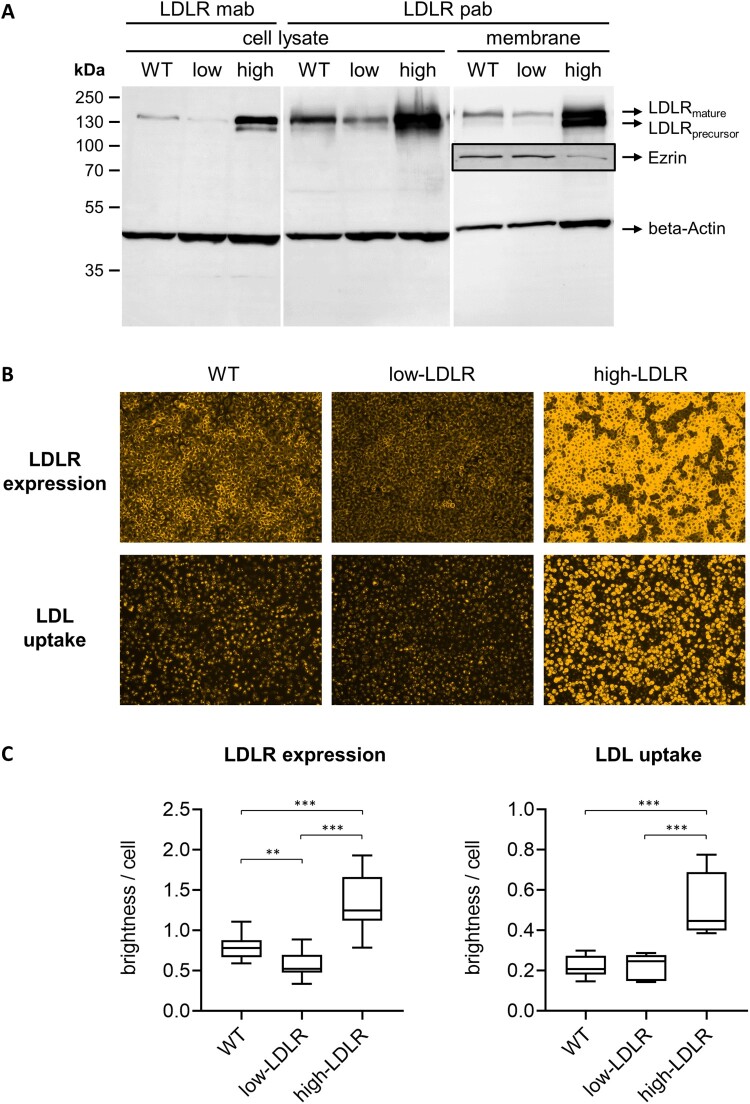
Phenotypic characterization of engineered embryonic porcine kidney cells (SPEV). (A) The LDLR expression of SPEV wild type (WT), CRISPR/Cas9-edited low-LDLR (low) and trans-complemented high-LDLR (high) cells was analyzed by Western Blot. LDLR in cell lysates and membrane protein preparations (membrane) was detected by either a monoclonal LDLR antibody (LDLR mab) or a polyclonal LDLR antibody (LDLR pab) in combination with a fluorescent secondary antibody. The housekeeping protein beta-Actin served for normalization. LDLR in membrane protein preparations was additionally quantified using the plasma membrane marker protein Ezrin (boxed, detected on a different blot with high-LDLR sample diluted 1:2). (B) SPEV WT, low-LDLR and high-LDLR cells were investigated for their total LDLR expression (top row) and LDL uptake (bottom row) by immunofluorescence (IF) microscopy. LDLR was stained after heat fixation, using the LDLR mab in combination with anti-rabbit secondary mab (orange). For analysis of LDL uptake, cells were deprived of FBS for 4 h before addition of fluorescent-labelled DiI-LDL (orange) for 1 h. (C) LDLR expression and LDL uptake were quantified by measuring the brightness of IF pictures using the ImageJ software and normalization against cell numbers. For each cell line, five pictures were taken and the experiments were repeated three times. Standard deviations are indicated. Significance of differences in LDLR expression was calculated by Brown-Forsythe and Welch ANOVA tests with Dunnett's T3 multiple comparisons correction and significance of differences in LDL uptake was calculated by Kruskal-Wallis test with Dunn's multiple comparisons correction (*p < 0.05; **p < 0.01; ***p < 0.001).

The LDLR expression of the two engineered porcine cell lines was analyzed by Western Blot and IF microscopy (Figure 3). Western Blotting showed that the LDLR expression levels of SPEV WT, the CRISPR/Cas9 edited and the trans-complemented cell line clearly differed from each other. Quantification of LDLR in cell lysates using the polyclonal LDLR antibody and beta-Actin for normalization revealed 48% lower or 60% higher LDLR expression in the two engineered cell lines. LDLR amounts in membrane preparations showed the same trends (50% less LDLR in the CRISPR/Cas9 edited cell line and 100% more LDLR in the trans-complemented cell line normalizing against beta-Actin, 42% less and 830% more LDLR normalizing against Ezrin) (Figure 3(A)). In IF microscopy, the CRISPR/Cas9 edited cell line appeared darker than the WT cells, indicating a weaker LDLR expression (**p < 0.01). In contrast, the trans-complemented cell line gave a considerably more intense signal (***p < 0.001) than the WT cells, revealing a stable and strong LDLR-overexpression (Figure 3(B+C)). Taken together, Western Blot and IF analysis provided consistent results, based on which the engineered SPEV cell lines were designated “low-LDLR” and “high-LDLR”.

#### Low-density lipoprotein uptake

In order to assess the functional activity of LDLR in the modified cell lines, cells were provided with fluorescent-labelled LDL and LDL uptake was monitored by IF microscopy (Figure 3(B+C)). Low-LDLR cells appeared slightly darker and high-LDLR cells appeared considerably brighter than the WT cells, indicating differences in the uptake of fluorescent-labelled LDL and further that the functional uptake of LDL corresponds well to the LDLR expression of the genetically modified cells. Quantitative analysis revealed no statistically significant reduction of LDL uptake in the low-LDLR cells, indicating that LDL uptake is only slightly reduced in comparison to the WT cells. In contrast, strongly enhanced LDL uptake (***p < 0.001) was confirmed for the high-LDLR cell line (Figure 3(B+C)). It can be concluded that the functional impact of the genetic modifications on LDL uptake is limited in the low-LDLR cell line compared to the prominent changes detected in the high-LDLR cells.

### Impact of cellular LDLR expression on infection with porcine pestiviruses

**Figure 4.**
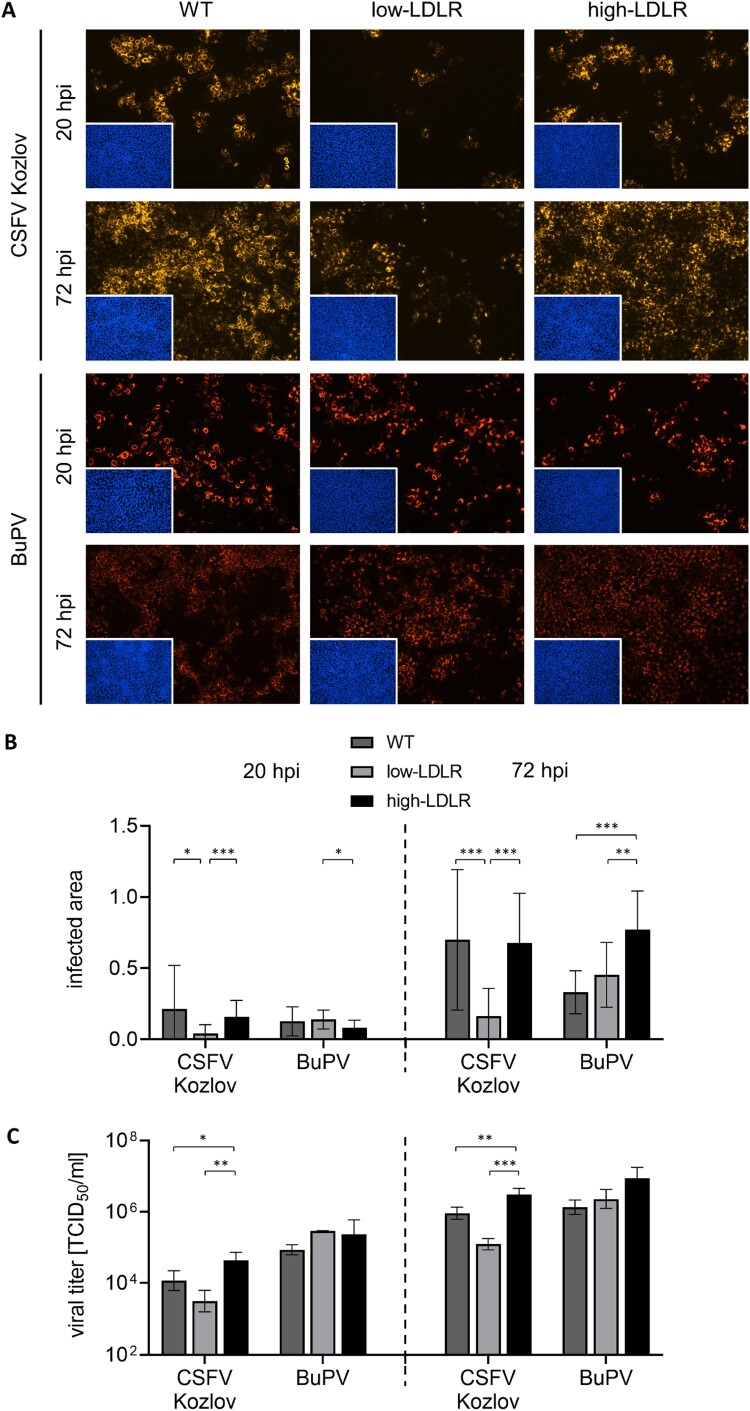
Impact of different LDLR expression levels on infection with porcine pestiviruses. (A) SPEV WT, low-LDLR and high-LDLR cells were infected with CSFV strain Kozlov and BuPV. Infections were evaluated at 20 and 72 hpi by immunofluorescence (IF) staining of pestiviruses using mab C16 for CSFV or porcine BuPV-specific antiserum, respectively, in combination with either anti-mouse (orange) or anti-swine (red) secondary mab. Nuclei were stained with DAPI (blue) to confirm the presence of confluent monolayers (small pictures in lower left corners). Representative pictures from three independent experiments are shown. (B) Infections were quantified as infected areas in IF by pixel counting using ImageJ. Orange/red pixels (detected in virus-positive cells) were normalized against blue pixels (detected in cell nuclei). Bars represent mean values from 15 pictures comprising five pictures from each of the three independent experiments, respectively. Standard deviations are indicated. Significance was calculated by Kruskal-Wallis test with Dunn's multiple comparisons correction (*p < 0.05, **p < 0.01, ***p < 0.001). (C) Infectious viral titers in the supernatants harvested from the infection experiments at 20 and 72 hpi were determined by endpoint dilution assay in quadruplicates. Bars represent mean 50% tissue culture infectious doses per ml (TCID_50_/ml) from three independent experiments. Standard deviations are indicated. Significance was calculated by ordinary one-way ANOVAs with Tukey’s multiple comparisons correction (*p < 0.05, **p < 0.01, ***p < 0.001).

Cell lines with different LDLR expression levels, namely SPEV WT and the generated low-LDLR and high-LDLR cells were infected with CSFV strains AlfT, Riems and Kozlov, and BuPV. In order to possibly obtain an indication of the stage of the viral replication cycle at which LDLR plays a role, infections were evaluated at two different time points. To identify effects likely related to initial steps in the replication cycle, an early time point after infection (20 hpi) was chosen, and to identify effects that may become evident only after multiple replication cycles, a late time point (72 hpi) was investigated (Figure 4, Figure S3).

For CSFV, IF microscopy and quantitative analysis of IF pictures revealed a strongly reduced permissivity of the low-LDLR cell line in comparison to the WT cells, which was most prominent for strain Kozlov (*p < 0.05 at 20 hpi; ***p < 0.001 at 72 hpi). This loss-of-function could be rescued by trans-complementation with LDLR, as demonstrated by the restored permissivity of the high-LDLR cells in comparison to the low-LDLR cells (***p < 0.001) (Figure 4(A+B)). The effects revealed by IF analysis were confirmed using FC. According to FC, 44% of WT cells were infected at 20 hpi, while 36% of low-LDLR and 56% of high-LDLR cells were positive. At 72 hpi, 74% WT, 40% low-LDLR and 85% high-LDLR cells were infected (mean values from three independent experiments). More importantly, the observed differences in the percentage of cells infected with CSFV also affected the production of viral offspring. Infectious viral titers of CSFV strain Kozlov were 3.5-fold reduced on the low-LDLR cells in comparison to the WT cells at 20 hpi and 7.5-fold at 72 hpi. In contrast, titers obtained from the high-LDLR cells were significantly enhanced in comparison to the low-LDLR cells (12-fold, **p < 0.01 at 20 hpi; 25-fold, ***p < 0.001 at 72 hpi) and even to the WT cells (3.5-fold, *p < 0.05 at 20 hpi; 3.5-fold, **p < 0.01 at 72 hpi) (Figure 4(C)). In general, the effects seen on the low-LDLR cells were less pronounced than the effects seen by antibody-mediated blocking of LDLR, indicating the latter being highly efficient. Results were most prominent for CSFV strain Kozlov, but CSFV strains AlfT and Riems showed the same dependence on LDLR (Figure S3), demonstrating that the impact of LDLR on viral replication is independent of the CSFV strain and genotype.

For BuPV, no major differences were observed in the permissivity of the three cell lines and the viral titers determined at 20 hpi (Figure 4). At 72 hpi, IF analysis and titration of the supernatants from the infected cells showed slightly enhanced infection and increased viral titers on the high-LDLR cells compared to the WT cells (Figure 4(B+C)). However, this presumed gain-of-function of the LDLR-overexpressing high-LDLR cells was not matched by a loss-of-function of the low-LDLR cells in comparison to the WT cells. Moreover, the differences in the BuPV titers were not statistically significant at either time point (Figure 4(C)). Together with the data obtained at 20 hpi, the results for BuPV did not indicate a clear impact of LDLR availability on BuPV infection.

### Impact of soluble LDLR on infection with porcine pestiviruses

**Figure 5.**
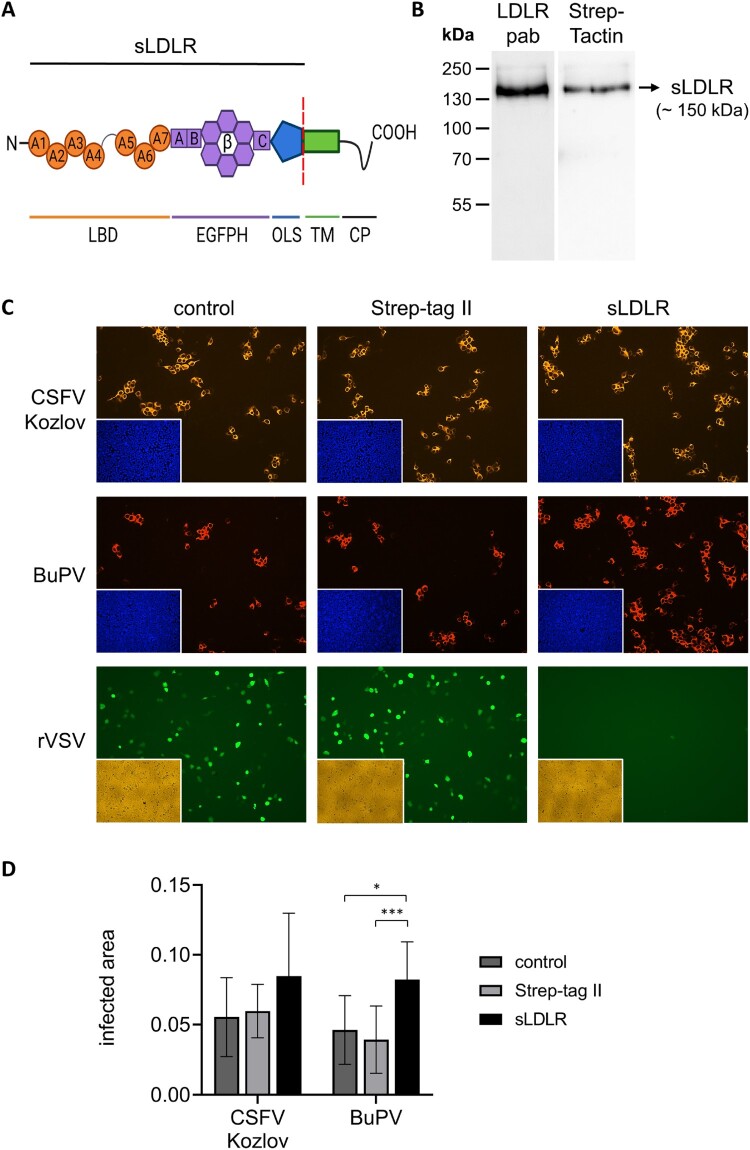
Impact of soluble LDLR on infection with porcine pestiviruses. (A) Recombinant soluble LDLR (sLDLR) represents the ectodomain of porcine LDLR consisting of the ligand binding domain (LBD), epidermal growth factor precursor homology domain (EGFPH) and O-linked sugar domain (OLS), lacking the transmembrane domain (TM) and cytoplasmic tail (CP) of native LDLR. TM and CP were replaced by a peptide containing a Twin Strep tag for affinity purification (not shown). Created with BioRender.com. (B) Purified fractions containing recombinant sLDLR were pooled and subjected to Western Blot analysis. sLDLR was detected using either a polyclonal LDLR antibody (LDLR pab) or a Strep-Tactin HRP conjugate (Strep-Tactin) binding to the Twin Strep tag. (C) CSFV strain Kozlov, BuPV and rVSV containing an eGFP-encoding sequence for green self-fluorescence were pre-incubated with either recombinant sLDLR, Strep tag control protein (Strep-tag II) or protein diluent (control) before infection of PK15 cells. After infection with CSFV and BuPV, the respective protein was added again for the incubation period of 20 h. Pestivirus infections were evaluated by immunofluorescence (IF) staining using mab C16 for CSFV or porcine BuPV-specific antiserum, respectively, in combination with either anti-mouse (orange) or anti-swine (red) secondary mab. Nuclei were stained with DAPI (blue) to confirm the presence of confluent monolayers (small pictures in lower left corners). rVSV infection of cells was monitored by eGFP expression (green) performing live cell imaging at 5 hpi. The presence of confluent monolayers was verified by bright field microscopy (yellow, small pictures in lower left corners). (D) CSFV and BuPV infections were quantified as infected areas in IF by pixel counting using ImageJ. Orange/red pixels (detected in virus-positive cells) were normalized against blue pixels (detected in cell nuclei). Bars represent mean values from 15 pictures comprising five pictures from each of the three independent experiments, respectively. Standard deviations are indicated. Significance was calculated by Kruskal Wallis test with Dunn's multiple comparisons correction (*p < 0.05, **p < 0.01, ***p < 0.001).

In order to further investigate the putative role of LDLR in entry of porcine pestiviruses, a decoy receptor assay was established. For this purpose, recombinant sLDLR was expressed in HEK 293 T cells as confirmed by Western Blot analysis. A specific band with a size of approx. 150 kDa representing sLDLR was observed using the polyclonal LDLR antibody or a Strep-Tactin HRP conjugate targeting the C-terminal Twin Strep tag (Figure 5(B)).

To confirm the integrity of the recombinant sLDLR and to control the experimental procedure, the decoy receptor assay was established with VSV, which reportedly uses LDLR as receptor [23]. As expected, the recombinant sLDLR led to almost complete neutralization of rVSV infection. In contrast to the neutralizing effect seen for rVSV, no significant effect on CSFV strain Kozlov infection was observed (Figure 5(C+D)). Surprisingly, the addition of recombinant sLDLR during BuPV infection resulted in a slightly increased infection in comparison to the infection control (78%, *p < 0.05) and to the specificity control (110%, ***p < 0.001) at 20 hpi. Strep tag peptides had no impact on infectivity (Figure 5(C+D)).

## Discussion

Since CSFV is a highly significant epizootic pathogen, many studies have addressed the interaction of CSFV with the porcine host cell. Several cellular factors involved in attachment, entry and later steps of the CSFV replication cycle have been described and recently reviewed [45-47]. However, despite the fact that there is increasing knowledge about the CSFV-host cell-interactome, many steps and details of the replication cycle of CSFV are still only poorly understood. Viral and cellular factors required for successful completion of the replication cycle of BuPV have not been investigated in detail. So far, there is only one study revealing that BuPV likely enters the host cell in a CD46-independent fashion like also seen for CSFV [17]. Considering the relevance of LDLR in infections with related viruses of the family *Flaviviridae* [24-27], this study set out to investigate the impact of LDLR in infection with the porcine pestiviruses CSFV and BuPV.

For a comprehensive analysis, loss-of-function and gain-of-function experiments were conducted. Antibody-mediated blocking of LDLR using PK15 and SPEV cells resulted in strongly reduced infections of both cell lines with all tested CSFV strains (CSFV strain Kozlov shown in Figure 1 and Figure S2, panel C). These results provide evidence for a general, cell line- and genotype-independent role of LDLR in CSFV replication. On BuPV infection, blocking of LDLR had a non-significant minor impact (Figure 1, Figure S2, panel C).

The relevance of LDLR in infection with CSFV and BuPV was further investigated using genetically modified cell lines. Although LDLR-KO has previously been successfully performed in human cell lines, CRISPR/Cas9-mediated genetic engineering of a dominant LDLR-negative porcine cell line was not possible within this study, probably due to a crucial role of LDLR in the cellular metabolism of the used porcine cell lines. Interestingly, a very recent study reported a successful LDLR-KO in porcine (PK15) cells [49]. Nevertheless, a characterization of the introduced genomic alterations and data demonstrating the absence of LDLR expression are lacking. In our study, positioning of the guide RNA within the sequence encoding for the SP resulted in a heterozygote SPEV cell line possessing one LDLR encoding allele that translates into an LDLR variant with a shortened SP and reduced expression at the plasma membrane (Figures 2 and 3).

The strong LDLR dependency of CSFV suggested by the blocking assay was confirmed using genetically modified SPEV cells. This observation was most prominent for CSFV strain Kozlov, a highly virulent strain that shows very efficient replication *in vitro* (Figure 4). BuPV replicates even more efficiently under *in vitro* conditions. Nevertheless, no or less clear effects of altered LDLR expression levels on BuPV replication were observed in the genetically engineered cell lines at 20 and 72 hpi, respectively (Figure 4). Thus, the much less pronounced reduction in permissivity to BuPV seen after antibody-mediated blocking of LDLR was not corroborated using cell lines with altered LDLR expression. These results indicate that LDLR is not an important molecular determinant in the viral replication of BuPV *in vitro,* in contrast to CSFV.

In addition to the liver, LDLR is expressed in all other investigated tissues of pigs, including heart, lung, spleen, kidney and ovary as well as peripheral blood mononuclear cells (PBMCs), preferential target cells of CSFV *in vivo* [49-52]. Interestingly, in humans, it was reported that activated and HCV-infected lymphocytes show increased LDLR expression levels, demonstrating a dynamic regulation of LDLR expression [53,54]. Although completion of the porcine LDLR expression profile is pending, it can be assumed that LDLR is widely expressed in various tissues of pigs, probably in all tissues that can be infected by CSFV. Thus, LDLR does not seem to be the decisive factor for CSFV’s tissue tropism. In this line, the also widely expressed human LDLR does not determine the strict tissue tropism of HCV [55]. Nevertheless, the relatively high LDLR expression in activated PBMCs and endothelial cells may favour CSFV replication.

In HCV infection, LDLR has been described to be involved in entry, but also in post-entry processes by contributing to the establishment of an optimized replication environment at intracellular membranes [26,56]. It is also involved in later stages of the replication cycle of other members of the family *Flaviviridae* mainly via regulation of cholesterol homeostasis [27]. Interestingly, upregulation of LDLR and changes in the cholesterol levels can be observed immediately after entry as early as 1 h – 6 h post infection with DENV [57]. A very recent study demonstrated that cholesterol biosynthesis modulated replication of CSFV strain Shimen [49]. Uptake of exogenous cholesterol via LDLR was blocked by CSFV-induced upregulation of proprotein convertase subtilisin/kexin type 9 (PCSK9) in PK15 cells. However, the data presented in this study did not provide evidence for a direct effect of LDLR depletion on replication of this CSFV strain [49]. Our data demonstrate for the first time that availability of LDLR is an important factor for CSFV replication. This is in line with a siRNA based investigation demonstrating that knockdown of LDLR expression had a significant impact on CSFV replication [18]. Thus, DENV and CSFV share their dependency on cholesterol as well as on LDLR. Interestingly, both viruses also can use the same attachment factors, namely heparan sulfate (HS) and the laminin receptor (LamR) [18].

One mechanism of HCV entry into hepatic cells can be via LDL-containing lipo-viro-particles that mediate interaction with LDLR. The fact that the low-LDLR cell line generated in this study showed only slightly reduced LDL uptake but strongly reduced permissivity to CSFV (Figures 3 and 4) might argue against LDL-mediated uptake. In previous studies, the question of direct interaction between LDLR and different virus species during cell entry has been addressed using recombinant soluble LDLR variants [23,26,58,59]. Applying the same strategy based on neutralizing properties of recombinant sLDLR, a decoy receptor assay was established to investigate the role of LDLR in entry of porcine pestiviruses. For CSFV, no evidence for a direct interaction with sLDLR was provided, while for BuPV, a slightly increased permissivity in the presence of recombinant sLDLR was observed (Figure 5). However, whether and how LDLR is involved in the replication cycle of BuPV remains to be investigated. More importantly, the lack of CSFV-neutralizing effects in the decoy receptor assay makes a major role of LDLR as a direct receptor for CSFV rather unlikely. For Japanese encephalitis virus (JEV), a member of the genus *Orthoflavivirus*, recent data suggest that LDLR might be a cofactor allowing internalization of the virus-receptor complex, instead of functioning as a direct JEV receptor [60]. LDLR may also contribute to CSFV uptake via a more unspecific way of internalization, such as caveolae-mediated endocytosis as recently suggested for severe acute respiratory syndrome coronavirus type 2 (SARS-CoV-2) [61]. Finally yet importantly, the functional involvement of LDLR in the life cycle of CSFV can be through influencing cholesterol uptake and lipid raft formation, being also important for entry as well as post-entry steps during replication of several other members within the family *Flaviviridae* (reviewed in [27]). Further studies are needed to unravel the exact function of LDLR in the replication cycle of CSFV.

Taken together, our results reveal a so far unknown relevance of LDLR during CSFV infection. In contrast, LDLR has no clear impact on the replication of the more distinct porcine pestivirus BuPV. The parallels between CSFV and other members of the *Flaviviridae* might allow the transfer of findings between different viruses of this family, offering interesting new research perspectives.

## Supplementary Material

Supplementary_Figures_revised_clean
