# Supplementary material for: Porcine low-density lipoprotein receptor plays an important role in classical swine fever virus infection
Source: Emerg Microbes Infect. 2024 Mar 21;13(1):2327385. doi: 10.1080/22221751.2024.2327385 (PMC10962300; doi:10.1080/22221751.2024.2327385)
Supplement: Supplementary_Figures_revised_clean [file TEMI_A_2327385_SM3903.docx]

**
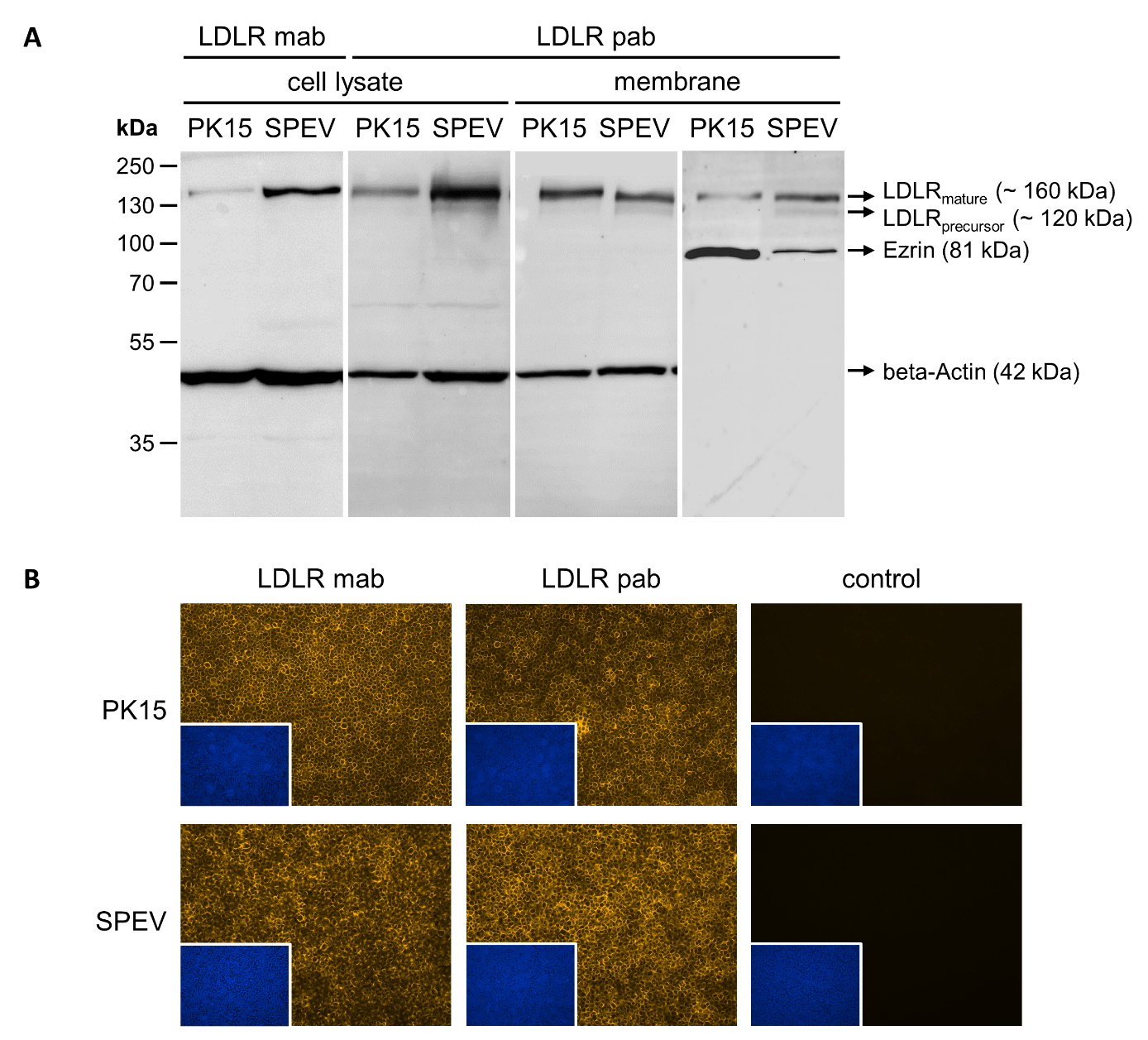
**

**Supplementary Figure S1.** Detection and expression of LDLR in porcine kidney cell lines PK15 and SPEV.

Reactivity of LDLR antibodies and LDLR expression of PK15 and SPEV cells were analyzed by (**A**) Western Blot and (**B**) immunofluorescence microscopy. (**A**) LDLR in cell lysates and membrane protein preparations (membrane) was detected by either a monoclonal LDLR antibody (LDLR mab) or a polyclonal LDLR antibody (LDLR pab) in combination with a fluorescent secondary antibody. The housekeeping protein beta-Actin served as loading control. Ezrin was used as a plasma membrane marker protein for the membrane protein preparations. (**B**) LDLR was detected by either the monoclonal or the polyclonal LDLR antibody in combination with Cy3-conjugated secondary antibody (orange). The specificity of the secondary antibody was controlled by omitting a first antibody (control). Nuclei were stained with DAPI (blue) to confirm the presence of confluent monolayers (small pictures in lower left corners).

**
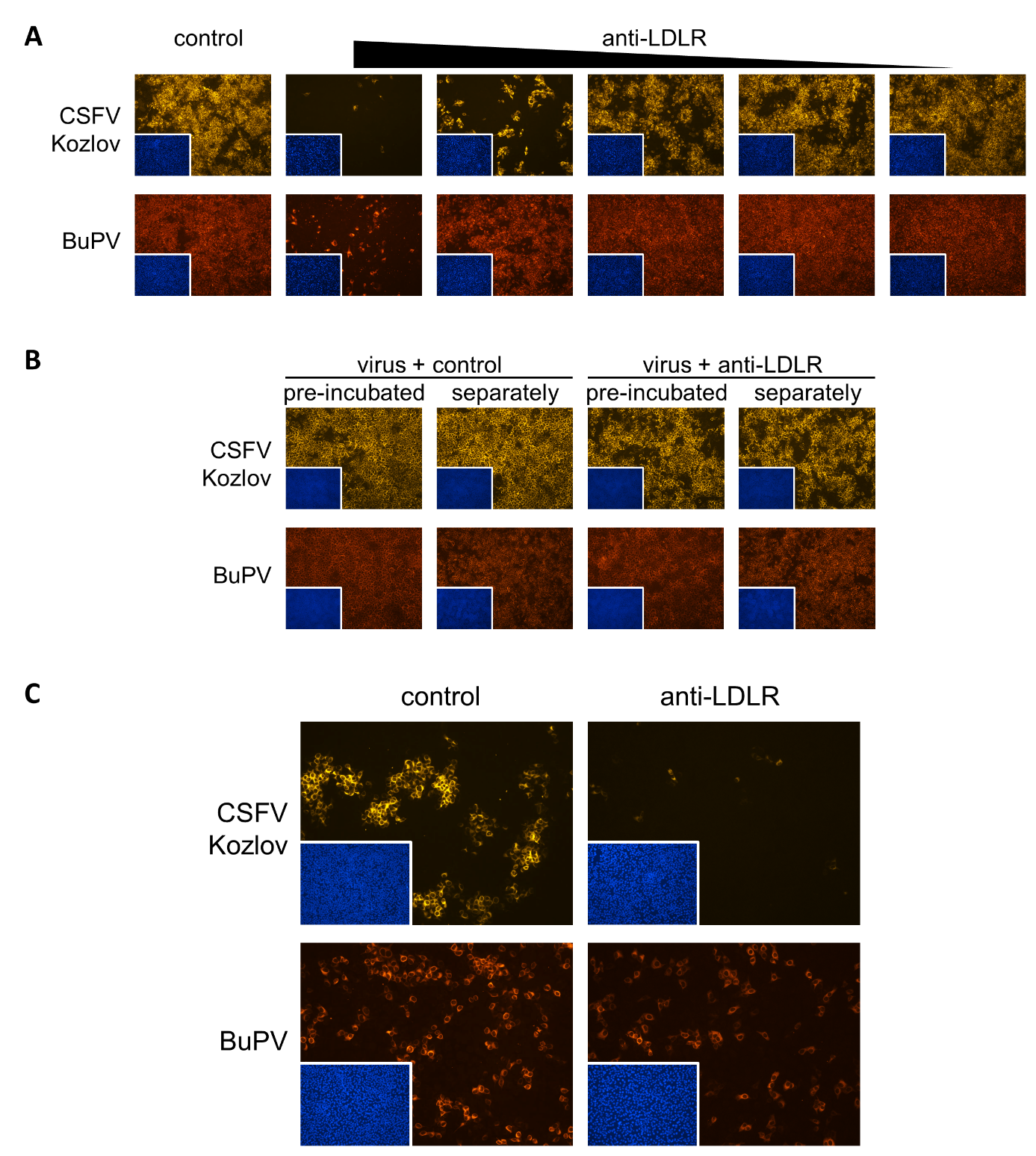
**

**Supplementary Figure S2.** Impact of LDLR-blocking on infection with porcine pestiviruses.

(**A**) PK15 cells were pre-incubated with either anti-LDLR antibody (anti-LDLR) in a 2-fold dilution series starting at 10 µg or 10 µg non-relevant anti-CD45 antibody (control) and infected with CSFV strain Kozlov or BuPV. After infection, the respective antibody and amount was added again for the incubation period of 20 h. (**B**) Virus inocula were pre-incubated with either 5 µg anti-LDLR or 5 µg control antibody before addition to PK15 cells or virus inocula and the respective antibody were separately added to the cells at the same time. The incubation period was 20 h. (**C**) SPEV cells were pre-incubated with either 5 µg anti-LDLR or 5 µg control antibody and infected with CSFV strain Kozlov or BuPV. After infection, the respective antibody was added again for the incubation period of 20 h. (**A-C**) Infections were evaluated by immunofluorescence staining of pestiviruses using mab C16 for CSFV or porcine BuPV-specific antiserum, respectively, in combination with either isotype-specific anti-mouse IgG1 (orange) or anti-swine (red) secondary mab. Nuclei were stained with DAPI (blue) to confirm the presence of confluent monolayers (small pictures in lower left corners). (**C**) Representative pictures from three independent experiments are shown.

**Supplementary Figure S3.** Impact of different LDLR expression levels on infection with Classical swine fever virus (CSFV) strains Alfort-Tübingen (AlfT) and Riems.

SPEV wild type (WT), low-LDLR and high-LDLR cells were infected with CSFV strains AlfT and Riems. Infections were evaluated at 20 and 72 hpi by IF staining of CSFV non-structural protein NS3 using mab C16 in combination with anti-mouse secondary mab (orange). Nuclei were stained with DAPI (blue) to confirm the presence of confluent monolayers (small pictures in lower left corners). Representative pictures from three independent experiments are shown. **
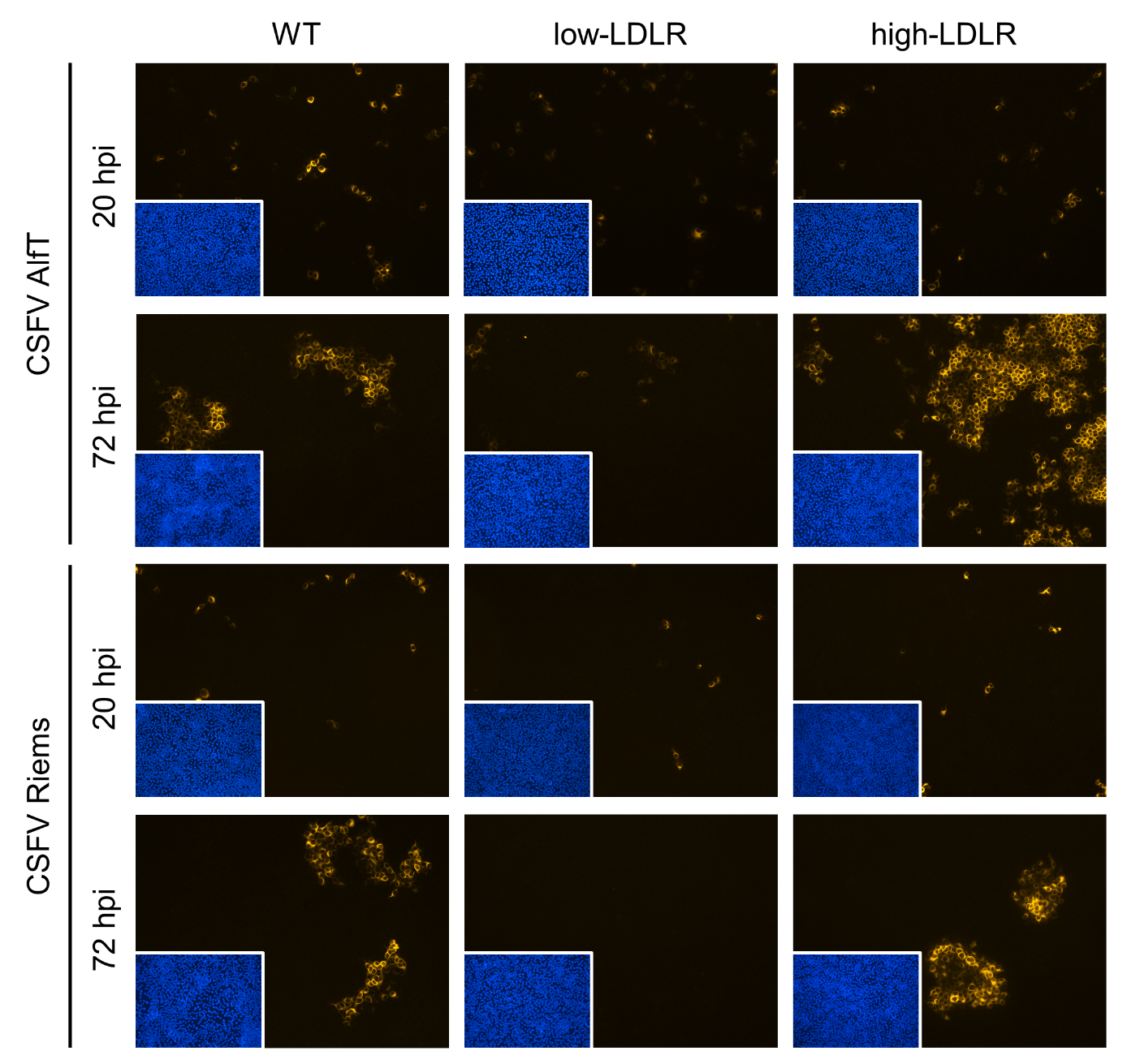
**
